# The impact of the traditional Chinese spring festival on glycemic control in a hospital population: a lag time-window analysis of 300,046 HbA1c tests

**DOI:** 10.3389/fendo.2026.1856168

**Published:** 2026-07-02

**Authors:** Dongmei Chen, Shiyu Deng, Chenglong Zhang, Ling Li

**Affiliations:** 1 Department of Health Management, West China School of Medicine, Sichuan University, Sichuan University affiliated Chengdu Second People’s Hospital, Chengdu Second People’s Hospital, Chengdu, Sichuan, China; 2 Department of International Medical Service, West China School of Medicine, Sichuan University, Sichuan University affiliated Chengdu Second People’s Hospital, Chengdu Second People’s Hospital, Chengdu, Sichuan, China; 3 Department of Laboratory, West China School of Medicine, Sichuan University, Sichuan University affiliated Chengdu Second People’s Hospital, Chengdu Second People’s Hospital, Chengdu, Sichuan, China; 4 Department of Nephrology, West China School of Medicine, Sichuan University, Sichuan University affiliated Chengdu Second People’s Hospital, Chengdu Second People’s Hospital, Chengdu, Sichuan, China

**Keywords:** Chinese Spring Festival, glycated hemoglobin (HbA) 1c, glycemic control, holiday, lag effect, real-world study

## Abstract

**Background:**

The Chinese Spring Festival involves marked lifestyle changes that may disrupt glycemic control. However, accurately quantifying its impact on long-term glycemic control measured by HbA1c is methodologically challenging because of the biomarker’s 2–3 month reflection period.

**Objective:**

This study aimed to quantify the adjusted association of the Spring Festival on HbA1c in a large hospital population by applying a novel “lag time-window” approach that accounts for the inherent lag of HbA1c.

**Methods:**

We analyzed 300,046 consecutive HbA1c tests from a tertiary hospital in China (2013–2025). The primary exposure was defined using a 60-day lag window: a test was considered exposed if the test date minus 60 days fell within the lunar Spring Festival week (New Year’s Eve to the 6th day). Nonparametric tests and quantile regression were used to adjust for age, gender, season, and department subgroup.

**Results:**

The median HbA1c during the lagged Spring Festival week was significantly higher than in the control period (6.1% vs. 6.0%; median difference +0.1%, P<0.001). The effect was most pronounced with the 60-day lag, attenuated at 30 days, and absent at 90 days. Subgroup analysis showed that the increase was concentrated in patients under diabetes core management (Festival week: 6.2% vs. control: 6.1%, P<0.05), with no effect in the general health screening population (P = 0.45). Quantile regression confirmed an independent association (β=0.041, 95% CI: 0.021–0.061, P<0.001).

**Conclusions:**

In this hospital-based population, after adjusting for the intrinsic lag of HbA1c, our findings suggest that the Spring Festival is associated with a small but statistically significant worsening of long-term glycemic control, specifically in high-risk patients with established dysglycemia. The pre-festival period should be recognized as a critical window for targeted intervention.

## Highlights

What are the main findings?

- This study introduces a novel “lag time-window” analytical method to accurately attribute the effect of the brief Chinese Spring Festival on HbA1c, overcoming a key methodological limitation related to the biomarker’s 2–3-month reflection period.
- In over 300,000 tests, the Festival was associated with a statistically significant increase in median HbA1c (+0.1%), an effect concentrated in high-risk patients under diabetes specialty care.

What are the implications of the main findings?

- The findings pinpoint the pre-festival period as a critical intervention window for proactive patient education.
- The study advocates for risk-stratified management strategies to mitigate holiday-associated glycemic excursions, particularly in vulnerable populations with established dysglycemia.

## 1 Background

Diabetes mellitus is a major public health challenge in China. Glycated hemoglobin (HbA1c), the gold standard for assessing long-term (2–3 months) glycemic control, is a critical predictor of diabetic complications (1). The traditional Chinese Spring Festival, the most important cultural holiday, involves unique customs such as family feasts, consumption of energy-dense foods, altered daily routines, and reduced physical activity, all of which may adversely affect glycemic control (2).

Emerging research, including studies on hypertension and acute cardiovascular events, has begun to characterize the Spring Festival as a period of systemic health risk, likely mediated by lifestyle disruptions (3, 4). Parallel work within diabetes has documented acute glycemic deterioration during the festival using continuous glucose monitoring or fasting blood glucose (5, 6), highlighting it as a “metabolic risk window.”

However, a pivotal and unaddressed question remains: what is the impact of this culturally significant but behaviorally disruptive holiday on long-term glycemic control as measured by HbA1c? Accurately quantifying this is methodologically challenging due to the biomarker’s inherent 2–3 month lag, a confounder that previous large-scale studies have rarely addressed (7). This gap limits our understanding and ability to develop targeted interventions. Standard analytical approaches that compare HbA1c levels during or immediately after the festival ignore this lag, potentially biasing the effect estimate toward the null or obscuring the true temporal relationship.

This study directly addresses this gap and aligns with the Journal of Diabetes’s focus on the interplay between culture, lifestyle, and diabetes outcomes. By leveraging HbA1c data from over 300,000 hospital visits and employing a novel “lag time-window” analytical approach, we aim to provide the first robust, methodologically sound estimate of the Spring Festival’s adjusted association with long-term glycemic control in a real-world population, and to identify vulnerable patient subgroups for targeted care.

## 2 Methods

### 2.1 Study design and data source

This single-center retrospective observational study extracted all HbA1c test records from the hospital information system of a tertiary care hospital in China between January 1, 2013, and December 31, 2025. The study was approved by the Institutional Review Board (Approval #[KY] PJ2025416). Waiver of informed consent was granted due to the retrospective nature of the study.

### 2.2 Study population

#### 2.2.1 Inclusion criteria

Having at least one valid glycated hemoglobin (HbA1c) measurement during the study period; Documented visit purpose (outpatient/inpatient/emergency/screening) and department.

#### 2.2.2 Exclusion criteria

Missing or clinically implausible HbA1c values (< 3.0% or > 25%); Laboratory quality control records; Research protocol data; Incomplete demographic characteristics.

No exclusions were made based on clinical diagnosis (including gestational diabetes) to preserve the completeness and heterogeneity of the real-world hospital population.

### 2.3 Variable definitions

#### 2.3.1 Primary outcome variable

HbA1c value (%) measured by high-performance liquid chromatography.

#### 2.3.2 Primary exposure variable (lag time-window)

Recognizing that HbA1c reflects average glycemia over the preceding 2–3 months (8), we defined the exposure period as the “lagged Spring Festival week.” Specifically: Test Date - N days ∈ Lunar Spring Festival Week (New Year’s Eve to the 6th day). Here N is the lag period in days. The primary analysis used a 60-day lag to match the primary reflection period. Sensitivity analyses employed 30-day and 90-day lags. The Control Period included all dates not falling into any lagged holiday window. Other national holidays (e.g., National Day, Labor Day, Qingming Festival, Dragon Boat Festival, Mid−Autumn Festival) were not excluded from the control period, which may result in a conservative estimate of the Spring Festival effect (i.e., the true effect may be larger than observed).

#### 2.3.3 Covariates and subgroups

Data on age, gender, and season of testing were collected. Based on the clinical context of the visiting department patients were categorized into four subgroups: 1) Diabetes Core Management Departments (e.g. Endocrinology); 2) Diabetes Complications-Related Departments (e.g., Cardiology, Nephrology, Neurology); 3) Non-Diabetes Primary Complaint Departments; and 4) Health Screening Center. Because patients could undergo multiple HbA1c tests over the study period, the same individual might contribute observations to both the exposed and control periods (i.e., a repeated−measures design).

### 2.4 Statistical analysis

Continuous variables are presented as mean ± standard deviation or median (interquartile range) depending on their distribution. Categorical variables are presented as frequency (percentage). Due to the non-normal distribution of HbA1c data, group comparisons were performed using the non-parametric Mann-Whitney U test (two groups) or the Kruskal-Wallis test (multiple groups). To assess the independence of the Festival effect, quantile regression (median regression) was used to assess the adjusted association of the Festival period, adjusting for potential confounders, including age (continuous), gender (male as the reference), season (included as dummy variables with winter as the reference category), and department subgroup. All analyses were performed using Python 3.13.7. A p value < 0.05 was considered statistically significant.

## 3 Results

### 3.1 Data extraction and cleaning

A total of 300,608 records were initially extracted from the LIS (Laboratory Information System). After applying exclusion criteria:

193 quality control records;

74 research protocol records;

245 records with missing HbA1c values;

48 records with HbA1c = 0;

2 records with HbA1c ≥100.

The final analytical dataset comprised 300,046 valid records (99.8% of the original data).

### 3.2 Baseline characteristics of the study population

A total of 300,046 HbA1c tests were analyzed. The mean patient age was 59.9 ± 16.5 years, and 51.6% (n=154,832) were male. The overall median HbA1c was 6.0% (IQR: 5.5%–6.8%), ranging from 3.0% to 24.8%. Departmental distribution was as follows: Diabetes Core Management Departments 15.8% (n=47,382); Diabetes Complications-Related Departments 25.6% (n=76,741); Non-Diabetes Primary Complaint Departments 37.1% (n=111,293); and Health Screening Center 21.5% (n=64,630). **Table 1** summarizes the baseline characteristics.

**Table 1:** Baseline characteristics of the study cohort (N = 300,046).

Characteristic	Mean ± SD or n (%)
Age (years)	59.9 ± 16.5
Male Gender	154832 (51.6%)
Overall HbA1c (%)	6.0 (5.5 – 6.8)
Department group (Original)	
Diabetes Core Management	47,382 (15.8%)
Diabetes Complications	76,741 (25.6%)
Non-Diabetes Primary Complaint	111,293 (37.1%)
Health Screening	64,630 (21.5%)

SD, Standard Deviation.

### 3.3 Primary analysis of the spring festival effect

Using the 60-day lag analysis, the median HbA1c during the lagged Spring Festival week was 6.1% (IQR: 5.6%–7.0%; n = 36,191), which was significantly higher than the 6.0% (IQR: 5.5%–6.8%; n = 227,664) in the control period (median difference: +0.1%; P < 0.001) (**Table 2**). The effect size (Cliff’s delta) was 0.032, indicating a small but highly statistically significant effect. The median HbA1c during the pre-Festival “high-risk” period and the post-Festival “recovery” period were both 6.0%, showing no significant difference from the control period.

**Table 2:** Comparison of HbA1c levels across time periods (60-day lag).

Period (60-day lag applied)	N	Median HbA1c (%)	IQR (%)
Lagged Spring Festival Week	36191	6.1	5.6 – 7.0
Control Period	227664	6.0	5.5 – 6.8
P value (Mann-Whitney U test)	< 0.001		

In descriptive analyses of time windows relative to the Spring Festival date, the highest median HbA1c values were observed during the festival week (6.4%, IQR: 5.8–7.5) and the first post-festival week (6.4%, IQR: 5.8–7.5), compared with 6.2% (IQR: 5.7–7.0) in the background period.

### 3.4 Sensitivity analysis and lag window validation

**Figure 1:**
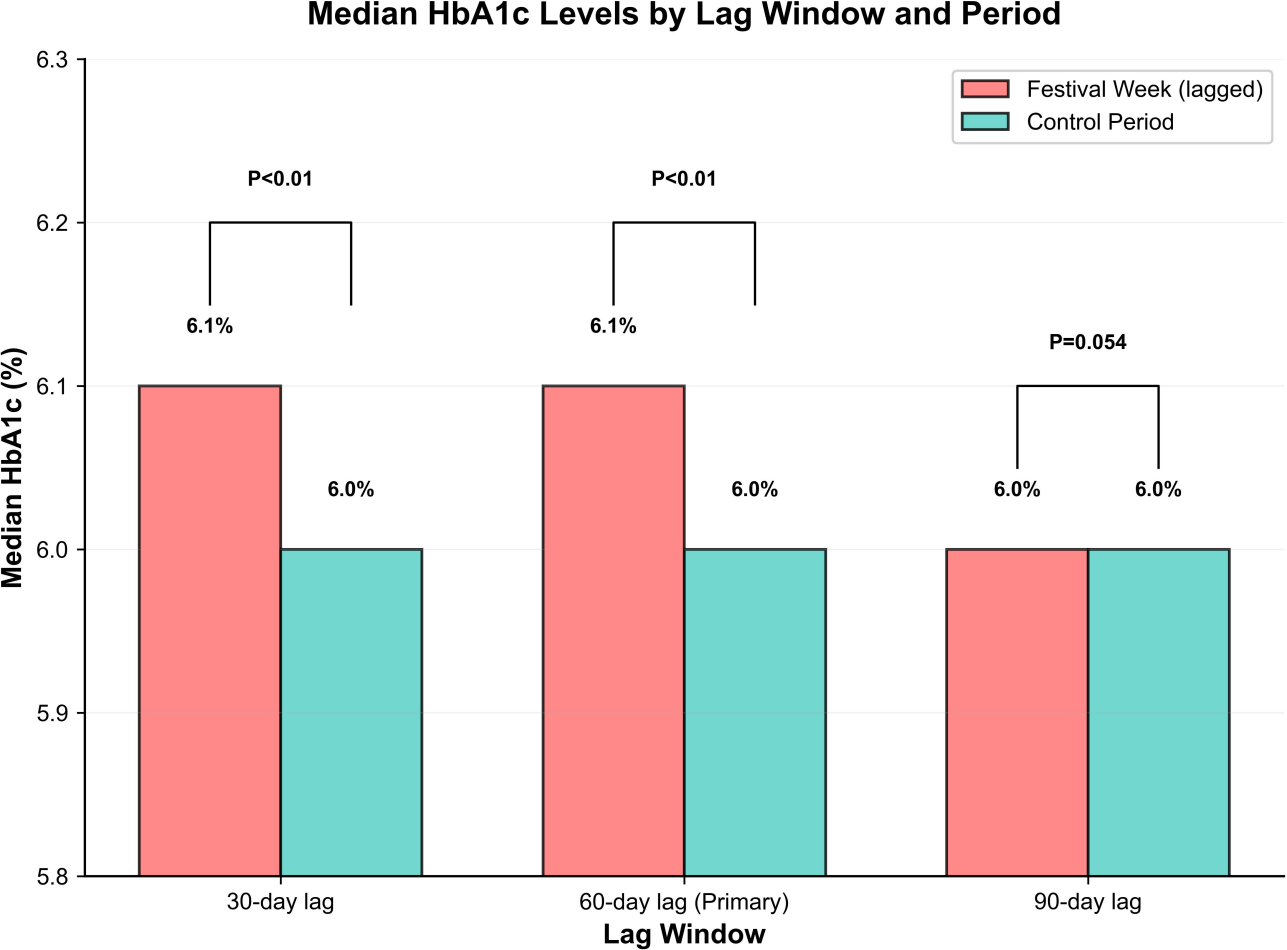
Comparison of median HbA1c levels during the lagged Spring Festival week versus the control period across different lag windows. Bar chart showing the median HbA1c (%) for tests where the date minus the lag period (30, 60, or 90 days) fell within the lunar Spring Festival week (“Festival”) compared to all other dates (“Control”). Error bars represent the interquartile range (IQR). The absolute median difference between the Festival and Control groups at the 60-day lag was 0.1%. The primary analysis used a 60-day lag period. Statistical significance was assessed using the Mann-Whitney U test (*P < 0.05, ***P < 0.001). ns, not significant.

Sensitivity analysis demonstrated that the Spring Festival effect was most pronounced and significant with the 60-day lag (median difference: +0.1%, P<0.001). The effect was attenuated with a 30-day lag (P = 0.002) and disappeared with a 90-day lag (P = 0.054). This pattern aligns with the physiology that HbA1c primarily reflects glycemia from the preceding 2 months, validating the rationale for selecting the 60-day lag window in our primary analysis (**Figure 1**).

Sensitivity analysis excluding other national holidays (clean control period). To test whether the inclusion of other national holidays in the control period biased the results, we re-defined a “clean” control period that excluded all other major national holidays (National Day, Labor Day, Qingming Festival, Dragon Boat Festival, Mid-Autumn Festival) and their ±7-day windows, as well as the two weeks before and after the Spring Festival. The median HbA1c in this clean control period was 6.20% (n=75,826), compared with 6.30% in the exposed group (n=24,624), yielding a difference of 0.10% (P<0.001). This confirms that the Spring Festival effect is robust, though modestly attenuated when other holidays are removed (**Table 3**).

**Table 3:** Sensitivity analysis using a clean control period (excluding other national holidays and peri-festival windows).

Group	N	Median HbA1c (%)	IQR	P value*
Exposed (Spring Festival lag window)	24,624	6.30	(5.8–7.3)	<0.001
Clean control	75,826	6.20	(5.7–7.2)	

* Mann–Whitney U test.

Sensitivity analysis restricted to the COVID-19 pandemic period (2020–2022).

We repeated the analysis using only tests from the pandemic years 2020–2022. Among 33,081 control tests and 6,481 exposed tests, the median HbA1c was 6.00% in the control group and 6.30% in the exposed group (difference 0.30%; P<0.001). The effect was larger than in the full cohort, which may reflect persistent household-based dietary changes despite lockdowns, although the smaller sample size warrants cautious interpretation (**Table 4**).

**Table 4:** Sensitivity analysis restricted to the COVID-19 pandemic period (2020–2022).

Group	N	Median HbA1c (%)	IQR	P value*
Exposed (Spring Festival lag window)	6,481	6.30	(5.8–7.4)	<0.001
Control	33,081	6.00	(5.5–6.9)	

* Mann–Whitney U test.

### 3.5 Subgroup analysis

#### 3.5.1 Department subgroups

**Figure 2:**
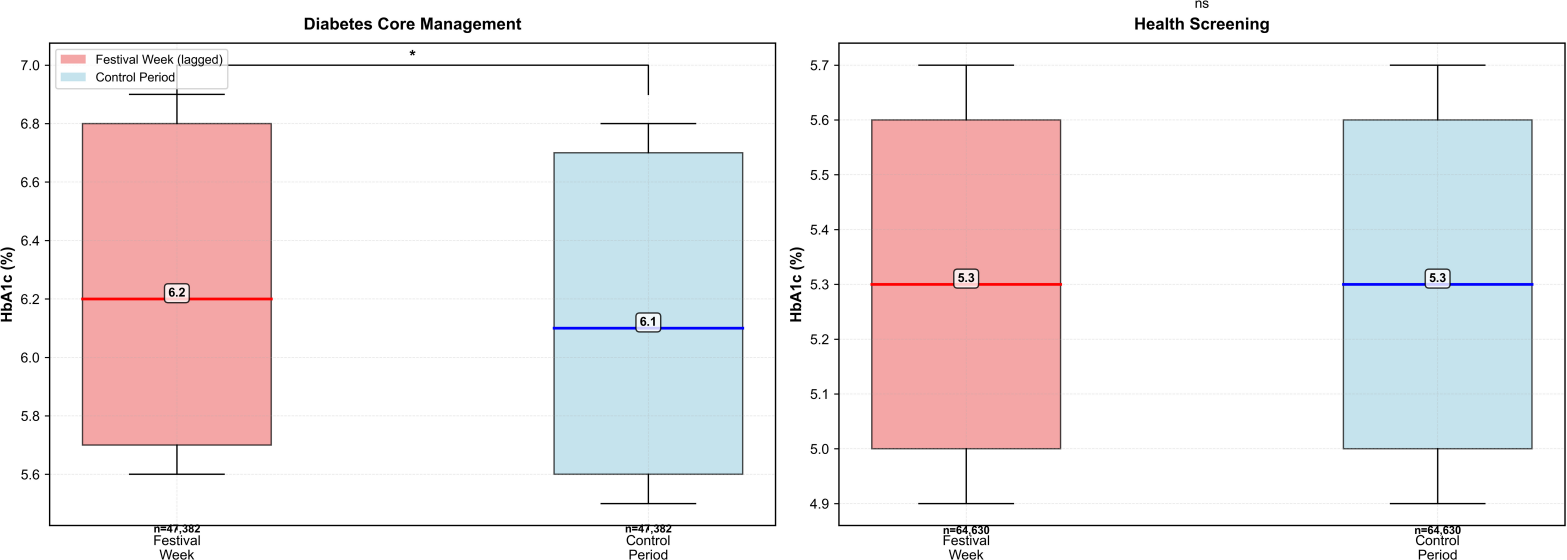
Subgroup analysis of the Spring Festival effect on median HbA1c across key department categories using the 60-day lag window. Comparison of median HbA1c (%) during the 60-day lagged Spring Festival week versus the control period within two representative subgroups: patients from Diabetes Core Management Departments (e.g., Endocrinology) and individuals attending the Health Screening Center. Bars indicate median values. The festival-associated increase was statistically significant in the Diabetes Core Management subgroup (P < 0.05, Mann-Whitney U test) but not in the Health Screening subgroup (P = 0.45).

The HbA1c-elevating effect of the Festival was most evident in patients from Diabetes Core Management Departments (Festival week: 6.2% vs. Control: 6.1%; P < 0.05). In contrast, within the Health Screening Center population, the median HbA1c was identical (5.3%) during the Festival week and the control period, showing no significant effect (P = 0.45) (**Figure 2**).

#### 3.5.2 Age and gender

No significant effect modification by age group (<40 40–60 60–80 >80 years) or gender was observed in the overall population.

### 3.6 Regression analysis

Quantile regression confirmed that the Spring Festival period (60-day lag) showed an adjusted association with higher HbA1c levels after adjustment for age, gender, department, and season (β = 0.041, 95% CI: 0.021–0.061, P < 0.001; **Table 5**). As a sensitivity analysis accounting for repeated measurements within individuals, we fitted a linear mixed−effects model with a random patient intercept. The Spring Festival lag window remained significantly associated with higher HbA1c (β = 0.027, 95% CI: 0.006–0.048, p = 0.012), consistent with the primary quantile regression.

**Table 5:** Factors associated with median HbA1c: results of multivariable quantile regression (median regression).

Predictor	Coefficient (β)	95% CI	P value
Spring Festival (60-day lag)	0.041	0.021 to 0.061	< 0.001
Age (per year)	0.014	0.013 to 0.014	< 0.001
Male Gender	0.284	0.272 to 0.295	< 0.001
Dept.: Diabetes Core (vs. Screening)	0.410	0.403 to 0.417	< 0.001
(Intercept)	5.631	5.605 to 5.656	< 0.001

### 3.7 Logistic regression for glycemic thresholds

To assess whether the Spring Festival lag window increased the risk of clinically relevant hyperglycemia, we performed binary logistic regression for HbA1c >7.0% and >8.0%, adjusting for age, gender, season, and department category. The odds ratios for exposure were 1.035 (95% CI: 0.996–1.076, p = 0.077) for HbA1c >7.0% and 1.043 (95% CI: 0.995–1.094, p = 0.078) for HbA1c >8.0%, indicating no statistically significant increase at these thresholds (**Table 6**).

**Table 6:** Logistic regression for HbA1c thresholds (>7.0% and >8.0%).

Outcome	Adjusted OR (95% CI)	P-value
HbA1c >7.0%	1.035 (0.996–1.076)	0.077
HbA1c >8.0%	1.043 (0.995–1.094)	0.078

Adjusted for age, gender, season, and department category (reference: Health Screening Center).

## 4 Discussion

### 4.1 Main findings and methodological implications

This large-scale, hospital-based real-world study provides robust evidence that the traditional Chinese Spring Festival is associated with a statistically significant, albeit modest, deterioration in long-term glycemic control (HbA1c +0.1%). The core innovation of our work lies in the application of a novel “lag time-window” analytical framework, which directly addresses the fundamental challenge of aligning a brief holiday exposure with the 2–3-month reflection period of HbA1c (8). The observation that the effect peaked with a 60-day lag, attenuated at 30 days, and disappeared at 90 days serves as compelling internal validation of both our methodological rationale and the biological plausibility of the finding. The biological rationale for detecting a short-term (7-day) lifestyle disruption using HbA1c lies in the weighted-average nature of the glycation process. HbA1c reflects mean glucose over the preceding 60–90 days, with approximately 50% of the final value determined by the most recent 30 days and about 25% by the last 10 days (10, 11). Consequently, a 7-day glycemic excursion during the Spring Festival can produce a small but statistically measurable shift in HbA1c, especially in large cohorts (12). This approach provides a replicable model for accurately quantifying the impact of any short-term lifestyle disturbance on HbA1c, moving beyond mere association to more credible temporal attribution.

Critically, the effect was not uniform but followed a clear risk gradient. It was concentrated entirely within patients under active management in Diabetes Core Departments, while being absent in a general Health Screening population. This pattern underscores that the Festival acts as a metabolic stress test, unmasking vulnerability in individuals with established dysglycemia or diminished metabolic flexibility, while those with robust glucose homeostasis remain resilient. For clinicians, this translates to a critical, actionable insight: the pre-Festival period represents a stratifiable risk window for their patients with diabetes.

### 4.2 Interpretation in context of existing literature and clinical relevance

Our findings significantly advance the understanding of holiday-associated metabolic risk. While previous studies, including those on other cardiovascular endpoints (3, 4), have established the Spring Festival as a general health stressor, and diabetes-specific research has shown acute glucose fluctuations (5, 6), our study provides a critical missing link. By employing the gold-standard long-term metric (HbA1c) and a novel methodology to account for its physiological lag, we demonstrate that the holiday’s disruption translates into a statistically significant deterioration in chronic glycemic control.

The effect size we observed (+0.1%) is consistent in magnitude with reports of HbA1c fluctuations around Western holidays (e.g., Christmas and New Year) (9) and during Ramadan (13), suggesting a commonality in the metabolic challenge posed by major cultural celebrations worldwide. More importantly, the stark contrast in effect between high-risk diabetes patients and the healthy screening subgroup is profoundly informative. It decisively argues against the notion that the festival effect is a non-specific, population-wide phenomenon driven solely by season or climate. Instead, it underscores that the holiday acts as a metabolic amplifier, exposing vulnerability in those with pre-existing dysglycemia or reduced β-cell reserve, who are less able to compensate for dietary excesses and routine disruptions (2).

Although the median difference of 0.1% reached statistical significance due to the large sample size, its clinical relevance at the individual patient level is modest. The typical laboratory measurement variability for HbA1c is around ±0.1−0.2% (14), and the observed difference falls within this range. Therefore, while the Spring Festival is associated with a measurable population-level deterioration in glycemic control, the effect should not be overstated as a universal risk for every patient. Instead, it highlights a vulnerable window for targeted public health messaging, particularly for those with established dysglycemia.

### 4.3 Strengths and limitations

The primary strengths of this study are its unprecedented real-world sample size, the novel and validated lag-adjusted methodology, and the clinically insightful subgroup analysis that precisely identifies the at-risk population.

Several limitations must be acknowledged. First, the observational design cannot establish causality, and residual confounding (e.g., by unmeasured dietary details or medication adherence) is possible, though our adjustment for season and subgroup findings mitigate key concerns. Second, the single-center design may affect generalizability; our findings necessitate validation across diverse geographic and healthcare settings in China. This limitation, however, also presents a clear future direction: a multi-center study would not only verify robustness but also explore regional variations in festival customs and their metabolic impact. Third, while we quantify the “what” and “who,” we cannot delineate the specific “how”—the exact behavioral mediators (e.g., particular foods, alcohol, sleep disruption) responsible for the HbA1c rise. Unpacking these mechanisms is essential for designing effective interventions.

Additional unmeasured confounders include body mass index (BMI), diabetes type (type 1 vs. type 2), diabetes duration, detailed dietary intake, and medication adherence (15, 16). Individuals with higher BMI or longer disease duration may be more susceptible to holiday-induced glycemic excursions, whereas type 1 diabetes patients might be less affected due to more rigid insulin regimens. The absence of these variables could bias our estimates in unknown directions, although our adjustment for department category (e.g., endocrinology vs. screening) partially captures differences in disease severity and treatment intensity.

### 4.4 Implications and future directions

The findings have direct and actionable implications. For clinical practice, the pre-Festival period should be recognized as a critical window for proactive, risk-stratified intervention. Targeted education for patients under diabetes care should be prioritized, focusing on practical strategies for navigating holiday feasts, maintaining physical activity, and adhering to medication schedules. Healthcare systems could implement supportive measures such as sending reminder messages, scheduling pre-holiday consultations, or providing culturally tailored dietary guidance (17).

Future research should aim to: 1) Validate and generalize these findings in multi-center and community-based cohorts across diverse regions; 2) Elucidate mechanisms by employing mixed-methods approaches that combine objective biomarker data (e.g., continuous glucose monitoring) with detailed behavioral tracking to identify the specific lifestyle factors most detrimental to glycemic control; and 3) Design and test interventions, such as brief “Pre-Festival Tune-Up” programs, to evaluate their efficacy in mitigating this predictable holiday-associated glycemic excursion.

## 5 Conclusion

In conclusion, this lag time-window analysis of over 300,000 HbA1c tests suggests that the Chinese Spring Festival is associated with a small but statistically significant worsening of long-term glycemic control in this hospital population. The effect, though modest in magnitude, is clinically meaningful and specifically concentrated in high-risk individuals under diabetes specialty care. These findings underscore the Festival as a period of metabolic vulnerability for patients with dysglycemia and highlight the importance of integrating cultural and lifestyle factors into personalized diabetes management strategies.

## Data Availability

The dataset is not publicly available due to hospital data governance policies and patient privacy protection. Access to anonymized data may be granted only upon reasonable request to the corresponding author and subject to approval by the institutional ethics committee. Requests to access these datasets should be directed to DC Email: meidongchen@foxmail.com.
